# Risk Factors for Group B Streptococcus Colonization Among Pregnant Women in Korea

**DOI:** 10.4178/epih/e2011010

**Published:** 2011-11-11

**Authors:** Eun Ju Kim, Kwan Young Oh, Moon Young Kim, Yong Soo Seo, Jung-Hwan Shin, Young Rae Song, Jae-Hyug Yang, Betsy Foxman, Moran Ki

**Affiliations:** 1 Department of Preventive Medicine, Eulji University, Daejeon, Korea.; 2 Department of Obstetrics and Gynecology, Eulji University Hospital, Daejeon, Korea.; 3 Department of Obstetrics and Gynecology, Cheil General Hospital & Women's Healthcare Center, Kwandong University, Seoul, Korea.; 4 Department of Obstetrics and Gynecology, Eulji Hospital, Seoul, Korea.; 5 Department of Epidemiology, School of Public Health, University of Michigan, Ann Arbor, USA.

**Keywords:** Colonization, Pregnant women, Risk factors, Screening, *Streptococcus agalactiae*

## Abstract

**OBJECTIVES:**

To identify obstetric and maternal factors related to Group B Streptococcus (GBS) colonization in pregnant women in Korea.

**METHODS:**

The study was conducted between the years 2006-2008 in four hospitals, Cheil and Eulji hospital in Seoul, and Motae and Eulji hospital in Daejeon. We recruited 2,644 pregnant women between 35 to 37 weeks of gestation who had visited for antenatal care. Participants completed a questionnaire, and urine, vaginal and rectal specimens were obtained and cultured using selective broth media. After delivery, medical records were reviewed.

**RESULTS:**

GBS colonization was significantly associated with hospital, age group, education, frequency of pregnancy, and premature rupture of membranes (PROM, more than 18 hours). After adjustment for other variables, Cheil hospital (odds ratio [OR], 2.05; 95% confidence interval [CI], 1.20-3.52), and the first pregnancy (OR, 2.32; 95% CI, 1.12-4.81) remained significant. History of vaginitis showed marginal significance (OR, 1.50; 95% CI, 0.98-2.29).

**CONCLUSION:**

To prevent GBS infection of neonates, clinicians should be alert to the potentially higher risk of GBS colonization in pregnant women in their first pregnancy, and women with premature rupture of membranes (PROM) (18 hours+) or who have a history of vaginitis.

## INTRODUCTION

Group B streptococcus (GBS, *Streptococcus agalactiae*) was recognized as a major cause of neonatal sepsis and meningitis in the 1970s [1], but the first Korean case of GBS neonatal meningitis was not described until 1984. Since then, the number of reported cases of neonatal GBS disease in Korea has increased steadily [2].

Neonatal sepsis usually develops within 3 days after birth, and the most frequent causative organism is GBS [3]. The main source of neonatal infection is maternal genital tract colonization [4]. GBS is transmitted vertically during labor and delivery, and colonization occurs in up to 80% of neonates born to colonized mothers [5]. Maternal streptococcal colonization is also associated with increased risk of urinary tract infection and pregnancy complications, such as endometritis [3], chorioamnionitis [3,6], preterm birth and intrauterine death [7].

Asymptomatic colonization with GBS is common worldwide, and depending on the population, between 6.5% [8] and 43.6% [9] of pregnant women are colonized with GBS in the vagina or rectum. Maternal GBS colonization varies by population characteristics such as age, parity, socio-economic status, geographic location [10], presence of sexually transmitted diseases [11] and sexual behavior [12].

The known risk factors of GBS colonization are as follows; delivery at <37 weeks' gestation, intrapartum temperature ≥38.0℃, or rupture of membranes for ≥18 hours [13]. After conducting a large population-based study, the Centers for Disease Control and Prevention (CDC) of the United States currently recommends screening of all pregnant women for GBS between 35 and 37 weeks of gestation [14]. For those with cultures positive for GBS, intrapartum antibiotic prophylaxis (IAP) during labor is recommended. Since this strategy was implemented, there has been a 70% reduction in neonatal early-onset sepsis [15].

Despite declines in early-onset GBS infection in the United States, GBS is identified in 40% of early-onset neonatal sepsis and remains a leading cause of newborn morbidity and mortality [14,16]. However, other European countries, which do not have high GBS prevalence like USA, do not recommend screening of all pregnant women for GBS.

The incidence rate of clinically diagnosed neonatal sepsis in Korea of 30.4/1,000 live births [17] is significantly higher than the 1.8/1,000 reported for the USA [18]. What percentage of this is attributable to GBS is unknown, as GBS screening is not a standard of care in Korea.

In 2006-2008, the prevalence of GBS colonization among 2,624 pregnant women in Seoul and Daejeon in Korea was 8% [19]. Risk factors for GBS colonization in pregnant women have not been previously studied in Korea.

To identify risk factors for GBS colonization in pregnant women, we analyzed the data of 2,644 Korean pregnant women who had a screened GBS culture test at 35-37 weeks gestation.

## MATERIALS AND METHODS

### Subjects

All consenting pregnant women of 35-37 weeks of gestation seen in Daejeon, at the Eulji University Hospital or the Motae Obstetrics and Gynecology (OBGY) Clinic, or in Seoul, at the Eulji General Hospital or Cheil Women's Hospital between January 2006 and May 2008 who had routine prenatal testing were included.

GBS was cultured and identified in the Departments of Laboratory Medicine of the Eulji hospitals in Seoul and Daejeon and, for samples obtained at Cheil Hospital, in the Seoul Clinical Laboratory. The laboratory also carried out antimicrobial resistance testing. The Department of Preventive Medicine of Eulji University performed GBS laboratory work for the Motae OBGY Clinic in Daejeon. The Institutional Review Boards at the Eulji (04-08 and 06-25) and Cheil (SCH-IRB-2005-24) hospitals approved the study protocol. Written informed consent permitting use of the sample materials and medical records for research purposes was obtained from every study participant.

### Questionnaire & medical record

Participants completed a self-reported questionnaire, which included questions on weight and height before or after pregnancy, symptoms during last two weeks, past history, the number of prenatal examinations, state of health, the presence of antibiotics-taking, food intake during last two weeks, smoking, alcohol intake, education and household monthly income. We reviewed medical records following delivery to collect gravidity, complications during the pregnancy, delivery type, the presence of ruptured membranes, and duration of membrane rupture. Gravidity indicates the total number of times a woman has been pregnant, regardless of whether these pregnancies were carried to term. The current pregnancy was included in the count.

### GBS isolates

#### GBS collection

Physicians collected vaginal mucus or discharge with a swab from the vaginal introitus without inserting a speculum, and placed the swab into Stuart's transport medium. A swab was inserted through the anal sphincter, rotated two or three times, and placed into a separate container of transport medium. Urine samples were self-collected specimens of the first 20 mL of urine. All participating laboratories used the same protocols for sample collection, GBS incubation and identification. Media and reagents were purchased by the combined research team and distributed to each participating laboratory.

#### GBS culture

Todd-Hewitt broth supplemented either with gentamicin (8 µg/mL) and nalidixic acid (15 µg/mL), or with colistin (10 µg/mL) and nalidixic acid (15 µg/mL) was used to repress the growth of microorganisms other than GBS. Urine samples were centrifuged, and 1 mL of the sedimented sample was placed into the selective medium. Rectal and vaginal swabs were removed from the transport medium and used to inoculate the selective broth medium. Cultures were shaken three or four times to ensure adequate mixing of the analyte. The lids of the culture tubes were loosely closed and the cultures incubated, along with a negative control, for 18-24 hours at 35-37℃ in ambient air containing 5% CO_2_. If the medium in the tubes was still clear after 18-24 hours, the cultures were reincubated and inspected again at 48 hours. Specimens with evident bacterial growth were subcultured on plates containing sheep blood agar, that is, a tryptic soy agar with 5% defibrinated sheep blood (TSAII; KOMED Co., Sungnam, Korea).

#### GBS identification

We used a catalase test followed by a latex agglutination assay (Streptex; Murex Biotech Ltd., Dartford, England) to confirm the isolate was GBS.

### Statistical analysis

All statistical analyses were done using SPSS version 18.0 (SPSS Inc., Chicago, IL, USA). The relationship between GBS colonization and various risk factors were tested for statistical significance using the chi-square test, test for trend, and multiple logistic regression models.

## RESULTS

### GBS colonization by demographic characteristics

The overall prevalence of GBS colonization among pregnant women at 35-37 weeks of gestation was 8.3% (219/2,644). Women <25 years had higher rates (14.0%), as did women identified at the Cheil hospital (10.7%). GBS colonization increased significantly with increasing education (p=0.034), but monthly income was not associated with GBS colonization. Smoking, alcohol intake during pregnancy, body mass index (BMI) before pregnancy, and subjective health status during pregnancy also were not associated with GBS colonization (Table 1).

### GBS colonization by obstetric characteristics

Women with one or more children or a history of two or more abortions had a significantly lower prevalence of GBS colonization (p=0.045 and p=0.034, respectively). This is consistent with pregnancy history: higher gravidity was associated with lower prevalence of GBS colonization (p=0.009). Delivery type, having antibiotics before GBS screening, number of prenatal vaginal examinations, and vaginal sonography were not related to GBS colonization. Although the association with premature rupture of membranes (PROM) was not significant, membrane rupture of greater than 18 hours was marginally associated with increased prevalence of GBS colonization (p=0.079), (Table 2).

### GBS colonization by symptoms and diseases

GBS prevalence was not associated with self-reported history for upper respiratory or gastrointestinal symptoms during the last 2 weeks. Among complications occurring during the current pregnancy, only a history of urinary tract infection was significantly related to GBS colonization (p<0.001). Self-reported lifetime histories of selected infectious and non-infectious diseases were not significantly associated with GBS colonization (Table 3).

### GBS colonization by food intake frequency

Regarding the frequency of food intake during last two weeks before the GBS test, drinking milk six or more time was significantly associated with GBS colonization (p=0.043). However fruits, vegetables, egg, chicken, pork, beef, raw fish and cooked fish, were not significantly associated with GBS colonization (Table 4).

### Risk factors for GBS colonization after adjustment using a multiple logistic regression model

All variables significant in the univariate analysis were included in a multiple regression model. After adjustment for hospital, age, gravidity, history of vaginitis and cystitis, associations with education level and frequency of drinking milk were no longer statistically significant. History of urinary tract infection and the duration of PROM over 18 hours were not included in the model because the sample size of urinary tract infection was very small (n=15) and only PROM positives have a duration of PROM.

Following adjustment, Cheil hospital (OR, 2.05; 95% CI, 1.20-3.52) and the first pregnancy (OR, 2.32; 95% CI, 1.12-4.81) were significantly associated with GBS colonization (Motae hospital, which is a private clinic for obstetrics, does not record abortions, so the cases from this hospital were not included in multiple regression analysis.). The history of vaginitis and cystitis were marginally significant, however, vaginitis increased colonization and cystitis decreased colonization. Women under 25 years had a higher colonization rate compared to women 35 years and older, but women 25 to 29 years old had a significantly lower prevalence. An interaction between age group and gravidity was not statistically significant; the interaction term was not included in the final model (Table 5).

## DISCUSSION

Among 2,644 pregnant Korean women screened between 35-37 weeks gestation, GBS colonization was significantly higher in the first pregnancy, and in one hospital.

Women less than 25 years of age had a higher prevalence of colonization, but there was no linear trend with age, and younger age was not a significant risk factor following adjustment. An association between age and gravidity and GBS colonization has been reported in some studies but not others; several studies [20-22] found no significant differences in colonization rates by age or parity, while others report associations with increasing gravidity [23], young age [24] and lower parity [10,24,25].

In other countries, young maternal age is a risk factor for early-onset neonatal sepsis [14,24,26,27] and infants who are born of teenagers show higher rates of GBS diseases [26]. In this study, only 3.5% of study participants were less than 25 years old; therefore, if an association with maternal age exists in Korea, it is swamped by other variables in our dataset.

In contrast to other reports where GBS prevalence is higher among those with lower socioeconomic status [28] and education [10], we observed higher colonization rates among women who are socially advantaged with a higher education. However, these associations were not significant following adjustment because a higher education was related to younger age, primigravida, and Cheil hospital.

Risk factors for colonization by serotype of GBS were analyzed. After adjustment, Cheil hospital (OR, 7.51; 95% CI, 1.02-55.46) and a history of vaginitis (OR, 2.18; 95% CI, 1.00-4.75) were significantly associated with serotype V GBS colonization. For other serotypes, there were no significant variables in multiple logistic regression models (data not shown).

As the GBS is a common colonizer of the genital and rectal area, the ascent of GBS from the vagina to the amniotic fluid likely increases the risk of neonatal infection. Therefore, frequent vaginal exams [6,29] or vaginal sonogram might increase risk of PROM or preterm labor. In this study we found that a high frequency of vaginal sonography (3 times or more) significantly increased risk of PROM (OR, 1.49; 95% CI, 1.13-1.97) compared to having a sonogram 0-2 times (data not shown). However, this risk was not significantly increased among women colonized with GBS.

Even though PROM was not related to significantly higher GBS colonization rates, among PROM mothers, PROM of 18 hours or more duration was marginally associated with a high prevalence of GBS colonization (p=0.079).

Among the symptoms during last two weeks before test, there were no related symptoms found. Among diseases during pregnancy, urinary tract infection was shown as a significant risk factor. However, the prevalence of urinary tract infection was very low - 0.5% (15/2,644).

Among four participating hospitals, Cheil hospital showed high rates of GBS colonization, which was still significant even in a multiple regression analysis. Cheil hospital is well known as a maternity hospital, and has the greatest number of deliveries in Korea; some 8,000 women give birth there each year. We could not explain why the GBS colonization rate is high in Cheil hospital in this study. In comparisons of four hospitals, there were no significant differences, except GBS colonization rates.

In the U.S in 1996, CDC recommended the use of one of two prevention strategies for decreasing GBS disease: a screening-based method, where IAP is offered to women identified as GBS carriers through prenatal screening cultures collected at 35-37 weeks' gestation and to women who develop premature onset of labor or rupture of membranes at less than 37 weeks' gestation, and the other is the risk-based method, where IAP is provided to women who develop one or more risk conditions at the time of labor or membrane rupture [13]. Subsequently, in 2002, CDC recommended that all pregnant women be screened at 35-37 weeks' gestation for vaginal and rectal GBS colonization, and if the result of GBS culture is not known at the onset of labor, IAP should be administered to women with any of the following risk factors (gestation <37 weeks, duration of membrane rupture ≥18 hours, or a temperature of ≥38.0℃) [14]. In the revised guidelines of 2010, the principles were not changed [30].

In the current study, 45% of the pregnant women had one or two risk factors (<25 years, and the first pregnancy); the GBS colonization rate was 10.8% in risk group. However, the GBS colonization rate for the no-risk group was 7.3%, suggesting that a better understanding of GBS risk factors in Korea is in order before recommending a risk-based strategy.

## Figures and Tables

**Table 1:**
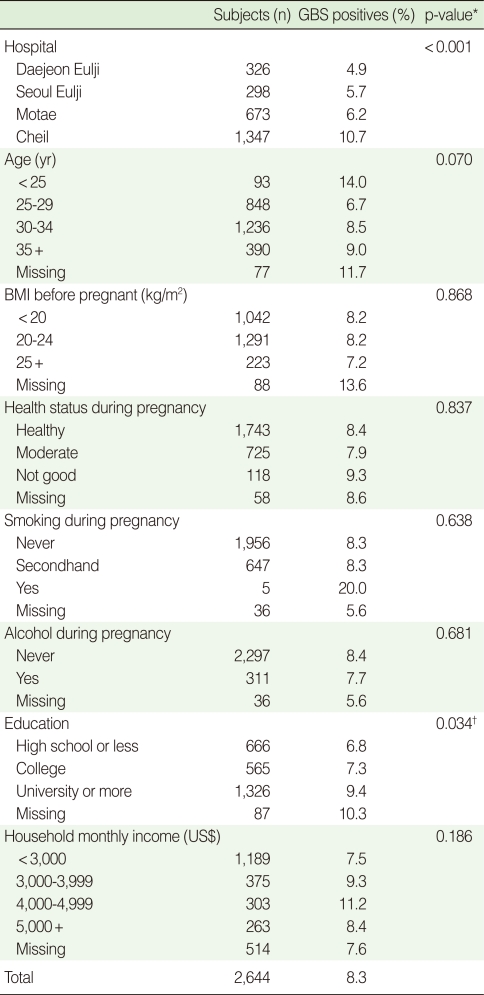
Group B streptococcus (GBS) colonization rate by demographic characteristics of pregnant women (35-37 weeks) in Korea (2006-2008)

BMI, body mass index. 1US$=1,000 Korea Won. ^*^p-value obtained by chi-square test; ^†^p-value obtained by trend test.

**Table 2:**
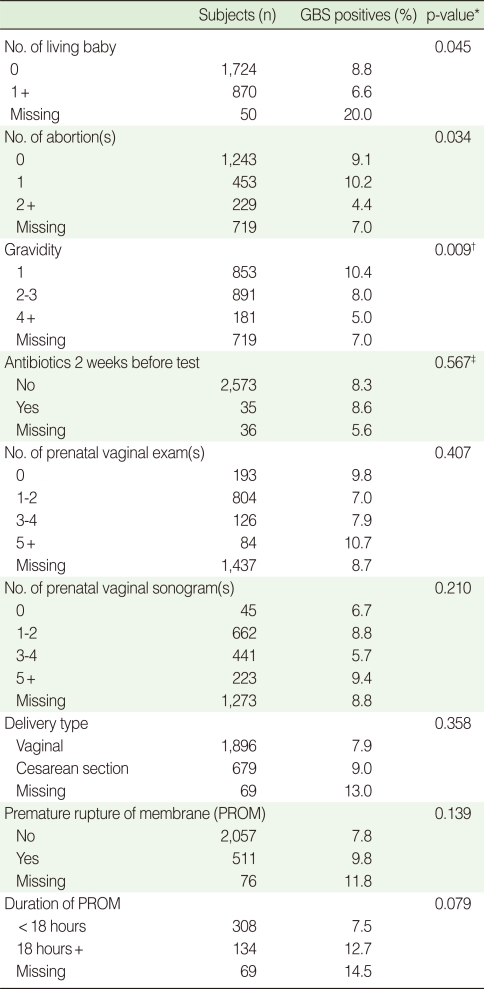
Group B streptococcus (GBS) colonization rate by obstetric characteristics of pregnant women (35-37 weeks) in Korea (2006-2008)

p-value obtained by ^*^chi-square test; ^†^trend test; ^‡^Fisher's exact test.

**Table 3:**
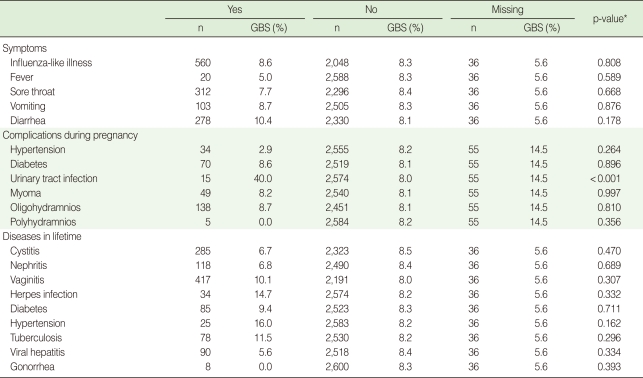
Group B streptococcus (GBS) colonization rate (%) by symptoms during last two weeks and diseases in pregnant women (35-37 weeks) in Korea (2006-2008)

^*^p-value obtained by chi-square test.

**Table 4:**
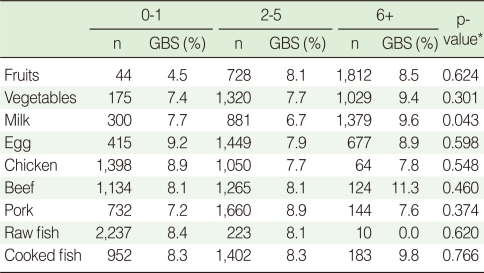
Group B streptococcus (GBS) colonization rate by frequency of food intake during last two weeks in pregnant women (35-37 weeks) in Korea (2006-2008)

^*^p-value obtained by chi-square test.

**Table 5:**
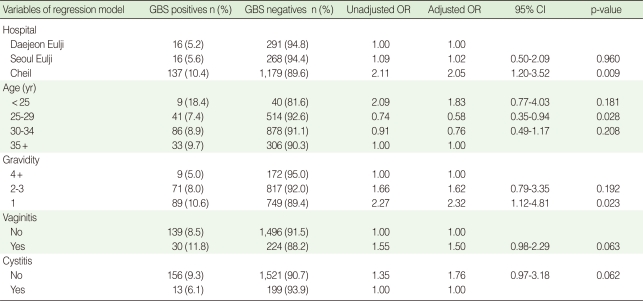
Risk of Group B streptococcus (GBS) colonization in pregnant women (35-37 weeks) in Korea (2006-2008) by using a multiple logistic regression model (n=1,907)
